# Human hepatocellular carcinoma cell-specific miRNAs reveal the differential expression of miR-24 and miR-27a in cirrhotic/non-cirrhotic HCC

**DOI:** 10.3892/ijo.2012.1716

**Published:** 2012-11-28

**Authors:** ALESSANDRO SALVI, EDOARDO ABENI, NAZARIO PORTOLANI, SERGIO BARLATI, GIUSEPPINA DE PETRO

**Affiliations:** 1 Department of Biomedical Sciences and Biotechnologies, Division of Biology and Genetics, University of Brescia, I-25123 Brescia, Italy; 2 Department of Medical and Surgical Sciences, University of Brescia, I-25123 Brescia, Italy

**Keywords:** human hepatocellular carcinoma, expression library, microRNAs, sequencing, bioinformatics

## Abstract

microRNAs (miRs) are 18–25 nucleotide non-coding RNAs that regulate gene expression in several physiological and pathological conditions. To gather more knowledge on microRNAs in human hepatocellular carcinoma (HCC) we generated a small RNA library in the human HCC cell line HA22T/VGH by cloning and sequencing the cDNA obtained following the size selection of 18–24 nucleotide RNAs. We determined the expression levels of the most frequently cloned microRNAs by qPCR in HCC tissues and in their peritumoral counterparts from biopsy specimens of 41 HCC patients. The most frequently cloned miRs were miR-24, miR-27a and miR-21, and their expression levels in human HCC tissues indicate that these miRs were dysregulated in HCC. We showed that miR-24 and miR-27a were significantly downregulated in HCCs from cirrhotic liver tissues in comparison to those from non-cirrhotic liver tissues. In cirrhotic HCCs the downregulation of miR-24 was correlated with poorer prognosis in patients with HBV and HCV virus infections. miR-21 was generally upregulated in HCC tissues *versus* the corresponding peritu-moral tissues, particularly in non-cirrhotic HCC. Furthermore, by sequence alignment we identified the human miR orthologue of *Mus musculus* miR-1199 not yet annotated. Our results outline the differential expression of miRs in cirrhotic and non-cirrhotic HCCs, thereby contributing to advances in the discovery and validation of novel molecular biomarkers of HCC progression.

## Introduction

microRNAs (miRNAs, miRs) are a class of small non-coding RNAs (18–25 nucleotides in length) that are widely expressed in the cells of several organisms such as invertebrates, vertebrates, plants and fungi. miRs typically bind to the 3′ untranslated region (UTR) of an mRNA target and direct the translational repression and/or degradation of this mRNA. miRNAs are involved in the control of many physiological processes, such as embryonic development, cell differentiation, proliferation and apoptosis. Additionally, miRs have been associated with several pathological conditions, including cancer, neurodegenerative diseases and autoimmune diseases (1–4).

To date, there are 2,042 annotated human mature miRs in the official registry (miRBase, http://microrna.sanger.ac.uk/sequences/) (5). Several authors suggest that additional miRs remain to be identified. Thus, the complete annotation of miRs is still an ongoing process. Many of the known miRs were discovered by examining miRNA libraries, whereby the cDNAs obtained following the size selection of 18–24 nucleotide RNAs were cloned and then sequenced (6,7). This method provided direct evidence for the existence and expression of miRs. miR libraries are also suitable for the determination of miR levels, which correlate with the cloning frequency. A limitation in the discovery of miRs by cloning is that it is difficult to find miRs that are expressed at a low level or only under specific conditions. This limitation could be overcome by deep sequencing, which allows for the detection of even poorly expressed miRs in the transcriptome as well as those not conserved across species (8). Further tools that aid in the search for new miRs are based on the bioinformatic prediction of miR sequences in the genomes (9–11). Briefly, these tools consider the conservation of the miR sequences across species and the intrinsic characteristics of their predicted hairpin structures such as the presence of a fold-back secondary structure and the thermodynamic stability of the hairpins; however, such computational methods do not predict whether a given miR is expressed in certain tissues or cells. Therefore, predicted miRs have to be experimentally validated (12).

Human hepatocellular carcinoma (HCC) is one of the cancers characterized by an extremely unfavorable prognosis and is the third leading cause of cancer-related death worldwide (13). In the present study, to gather novel information concerning miRs in HCC and with the aim to clone new miRs, we developed a small RNA expression library in the HCC cell line HA22T/VGH. We analyzed the expression profile of the miRs most frequently cloned (miR-24, miR-27a and miR-21) in the tumor and peritumoral tissues from biopsy specimens of patients presenting with HCC.

## Materials and methods

### Cell cultures

Undifferentiated HCC-derived cells, HA22T/VGH, were maintained in RPMI-1640 (Invitrogen, Carlsbad, CA, USA) supplemented with 10% fetal bovine serum at 37°C in a 5% CO_2_ incubator. These cells were kindly provided by Professor N. D’Alessandro (University of Palermo, Italy). SKHep1Clone3 (SKHep1C3) (14), selected from human HCC-derived cells (SKHep1, ATCC HTB-52) were maintained in Earle’s MEM (Invitrogen) supplemented with 10% fetal bovine serum (Invitrogen) at 37°C in a 5% CO_2_ incubator.

### Tissues and clinicopathological features of HCC

All of the human HCC samples (n=41) as well as the corresponding peritumoral (PT) non-tumor samples (resected 1–2 cm from the malignant tumor) were obtained from HCC patients for pathological examination. Each biopsy specimen was obtained following patient informed consent under standard conditions of sampling and processing as previously described (15). Each specimen was determined to be HCC or PT by pathological examination. In this study, 41 HCC subjects underwent surgical resection. The subjects consisted of 28 men and 13 women (40 Italian and 1 Chinese) ranging from 38 to 82 years of age (mean age, 67.9±8.9 years). The subjects did not have any apparent distant metastases, and none had been previously treated for HCC. The PT tissues revealed the presence of different background diseases (25 cirrhosis, 15 hepatitis, 1 steatosis), and the patients were analyzed for the presence of the hepatitis B (HBV) and hepatitis C (HCV) viruses. Fifteen patients were positive for HCV, 10 were positive for HBV, 4 were positive for both HBV and HCV and 7 were found to be negative for both HBV and HCV; for 5 patients no information was available (Table I).

### Small RNA isolation

Total RNA was extracted from HA22T/VGH cells using the miRNeasy Mini kit (Qiagen) and the small RNAs (<200 nucleotides) were further separated using the RNeasy MinElute Cleanup kit (Qiagen, Gaithersburg, MD, USA) according to the manufacturer’s instructions. Six full-confluence 10-cm plates of HA22T/VGH cells were used to extract the small RNAs.

### Construction and screening of a cDNA library of small RNAs

Small RNAs were polyadenylated at 37°C for 60 min in a 100-*μ*l reaction volume with 1.5 *μ*g of RNA and 8 U of poly(A) polymerase (Ambion, Austin, TX, USA). Poly(A)-tailed small RNAs were recovered using the miRNeasy Mini kit. A 5′ adapter (5′-CGA CUG GAG CAC GAG GAC ACU GAC AUG GAC UGA AGG AGU AGA AA-3′) was ligated to the poly(A)-tailed RNAs using T4 RNA ligase (Promega) in a 40-*μ*l reaction volume at 37°C for 30 min. The ligation products were recovered by the miRNeasy Mini kit. Reverse transcription was performed using 1.5 *μ*g RNA and 55 pmol of the RT primer (5′-GTA CAG CCG GCG GAG CCG GAG ATC TTA -d(T)30 (A, G, or C) (A, G, C, or T)-3′ with 200 U of SuperScript III reverse-transcriptase (Invitrogen). Poly(A)-tailed small RNAs (20 *μ*l total volume) were incubated with 0.55 *μ*l of the RT primer and 5 *μ*l of a dNTP mix (2 mM) at 65°C for 5 min to remove any RNA secondary structure. The reactions were chilled on ice for at least 1 min and the remaining reagents [5X buffer, dithiothreitol (DTT), RNaseout, SuperScript III] were added as specified in the SuperScript III protocol. The reaction was allowed to proceed for 60 min at 55°C. Finally, the reverse transcriptase was inactivated by a 15-min incubation at 70°C. The cDNA amplification was carried out for 30 cycles at a final annealing temperature of 50°C using the forward primer 5′-GGA CAC TGA CAT GGA CTG AAG GAG TA-3′ and the reverse primer 5′-ATT CTA GAG GCC GAG GCG GCC GAC ATG T-3′. The PCR was performed using GoTaq DNA polymerase (Promega, Madison, WI, USA). The PCR product was separated on a 2% agarose gel with ethidium bromide staining and gel slices containing ∼109 nucleotide-long DNA were excised. The DNA was purified using the Wizard SV^®^ Gel and PCR Clean-Up System kit (Promega). The DNA fragment was directly subcloned into the pGEM-T vector (Promega) which was subsequently used to transform JM109 competent cells (Promega). Colony PCR was performed using primers for the T7 promoter (5′-TAA TAC GAC TCA CTA TAG GG-3′) and SP6 promoter (5′-ATT TAG GTG ACA CTA TAG AA-3′) and the clones resulting in PCR products of ∼271 bp in length were sequenced using the ABI PRISM 310 Genetic Analyser (Applied Biosystems, Foster City, CA, USA).

### Real-time quantification of mature miR-24, miR-27a and miR-21 by stem-loop RT-PCR

Total RNA from tissue samples was isolated using the TRIzol reagent (Invitrogen) according to the manufacturer’s instructions. For a quantitative analysis of mature miRs, a two-step Taq-Man real-time PCR analysis was performed using primers and probes obtained from Applied Biosystems. cDNA was synthesized from total RNA (50 ng) in 15-*μ*l reactions using reverse transcriptase and the stem-loop primers for miR-24 (Applied Biosystems; assay ID 000402), miR-27a (assay ID 000408), miR-21 (assay ID 000397), or RNU66 (internal control; assay ID 001002) provided with the TaqMan MicroRNA Reverse Transcription kit (Applied Biosystems). The reverse transcriptase reaction was performed by incubating the samples at 16°C for 30 min, 42°C for 30 min, and 85°C for 5 min. Each PCR reaction (20 *μ*l) contained 1.3 *μ*l of reverse transcriptase product, 10 *μ*l of Taq-Man 2X Universal PCR Master Mix, and 1 *μ*l of the appropriate TaqMan MicroRNA Assay solution containing primers and probes for each miR of interest. The PCR mixtures were incubated at 95°C for 10 min followed by 40 cycles of 95°C for 15 sec and 60°C for 60 sec. PCRs were performed in triplicate using a 7500 Real-Time PCR system. The expression level calculations for the 3 miRNAs were based on the ΔΔC_T_ method, using RNU66 as an internal control. For each case the ratio between the relative levels in HCC and those in PT was assessed. The level of expression of the miRNAs was considered to be decreased for a value <0.7 and increased for a value >1.3. A value between 0.7 and 1.3 was defined as having no change in expression level.

### Bioinformatic analyses

The cloned RNA sequences were compared to the sequences in miRBase (http://www.mirbase.org/). Sequences not found in the miRNA database were subjected to BLAST (http://blast.ncbi.nlm.nih.gov/Blast.cgi) or BLAT (http://genome.ucsc.edu/) analyses against the human genome (http://www.ncbi.nlm.nih.gov/blast). The mFold Web server (http://mfold.rna.albany.edu/?q=mfold) was used to evaluate the ability of the candidate miRNA sequence to form thermodynamically stable hairpin structures. The tool Clustal W2 (http://www.ebi.ac.uk/Tools/msa/clustalw2/) was used to evaluate miRNA candidate sequence conservation between different species.

### Validation of the miRNA candidate sequence by northern blotting

The total RNA samples (7.5 *μ*g each) and the miR markers (New England Biolabs, UK) (17, 21 and 25 nucleotides in length) were electrophoresed on 15% TBE/urea precast gels (Invitrogen) and transferred to a Nylon^+^ membrane (Invitrogen). The hybridi zation buffer, wash buffer, blocking buffer and detection buffer were supplied by Signosis (Sunnyvale, CA, USA). Membranes were hybridized (42°C for 16 h) with 5′-biotin-labelled mercury LNA detection probes (Exiqon, Vedbaek, Denmark) corresponding to the complementary sequences of the mature miRNA candidate (hsa-miR-1199 probe: 5′-CTG CGC GGC CCG GGC TCA GG-3′, hsa-miR-1199^*^ probe: 5′-GTT GAG CAC CGG CCG CAC GC-3′). As a control, the blots were hybridized with an oligonucleotide probe complementary to the U6 RNA (U6 probe: 5′-CAC GAA TTT GCG TGT CAT CCT T-3′). The blots were incubated with streptavidin-coupled horseradish peroxidase (Signosis) and signals were detected by enhanced chemiluminescence (Thermo Scientific, Rockford, IL, USA). The results were visualized on X-ray film (Thermo Scientific).

### Statistical analysis

The histograms represent the mean values, and the bars indicate standard errors of the mean. The statistical significance of the results was determined using the Student’s t-test. The linear correlation between miR expression and overall survival was determined by the Pearson correlation test. The data were considered significant at p<0.05. The statistical analysis was performed with KyPlot (v.2.0b15, http://www.woundedmoon.org/win32/kyplot.html).

## Results

### miRNAs identified by a small RNA expression library in HA22T/VGH cells

**Figure 1.**
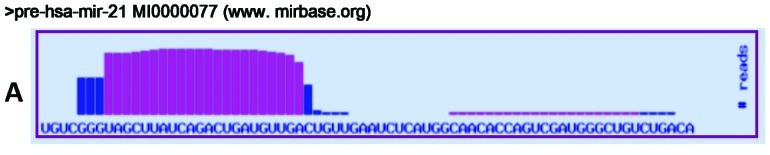

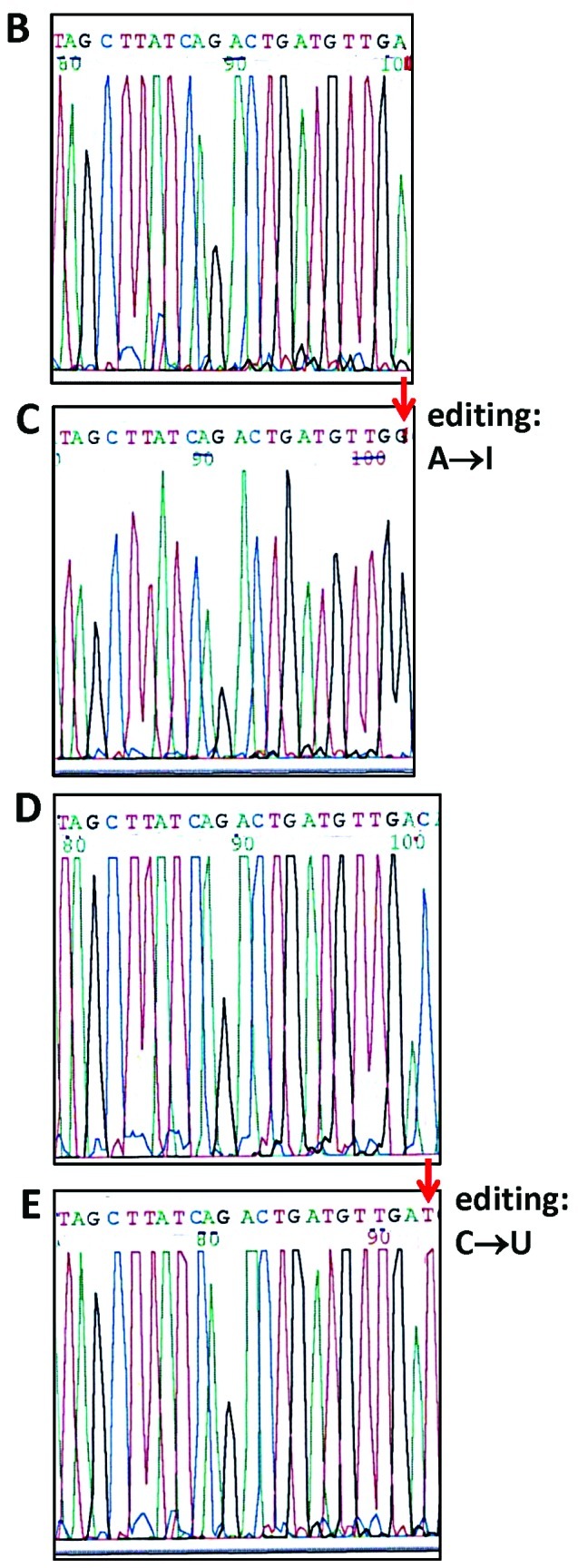
(A) hsa-pre-miR-21 sequence. (B and D) The most frequent miR-21 sequences found. The most frequent variations at the 3′ terminus of the mature miR-21 resulted from A→I editing (4/51; 7.8%) and C→U (5/51; 9.8%) (C and E respectively).

To uncover new miRNAs and to study global miR expression in HCC, a small RNA library was generated in the HCC cell line HA22T/VGH. A total of 200 bacterial clones were sequenced and 118 clones corresponded to 31 known miRNAs (Tables II and III). The most frequently isolated miRNAs were miR-24, miR-27a and miR-21 (Table III). For miR-21, we found sequence differences located at the 3′ end of the mature miR. The most frequent variations resulted from C→U (5/51; 9.8%) and A→I editing (4/51; 7.8%) (Fig. 1). Three clones corresponded to the precursor stem-loop of the hsa-miR-1308, and 1 clone was partially homologous to the mmu-miR-1199 precursor stem-loop. Twenty-five clones corresponded to rRNAs, tRNA, snRNAs, mitochondrial RNAs and mRNA fragments, 31 clones were empty and 19 clones could not be sequenced.

### miR-24, miR-27a and miR-21 differential expression in HCC tissues from human biopsy specimens

The expression levels of the most frequently cloned miRNAs, miR-24, miR-27a and miR-21, were evaluated using real-time PCR in the tumor and corresponding PT tissues from the biopsy specimens of 41 HCC patients. The ratio (R) was determined between the miR expression level (RQ_HCC_) in the HCC sample versus the expression level (RQ_PT_) detected in the PT. We assumed miR upregulation when R>1.3, miR downregulation when R<0.7 and no variation when 0.7≤R≤1.3.

**Figure 2.**
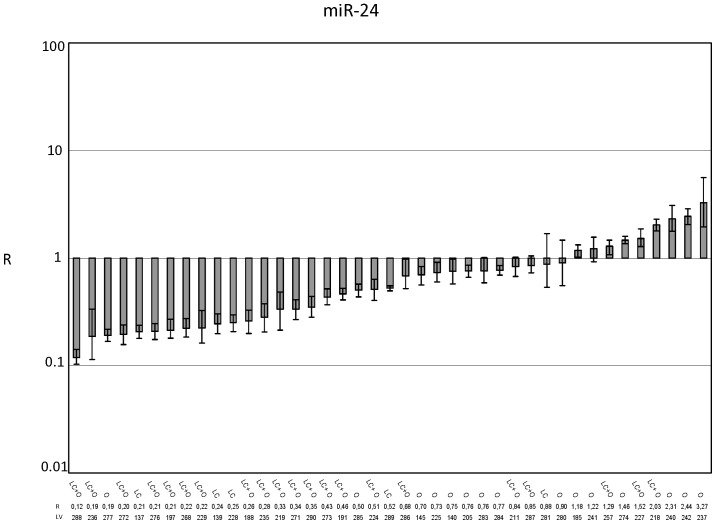
miR-24 expression levels detected by real-time PCR in tissues from biopsy specimens from patients affected by HCC. The graph indicates the R (RQ_HCC_/RQ_PT_) corresponding to the human sample tested. The histograms are ordinated by increasing R. The background diseases are also indicated (LC, liver cirrhosis; O, other background disease i.e., B/C viral hepatitis, steatosis). The R values and the case number (LV) are listed under the graph.

miR-24 (Fig. 2) was not dysregulated, based on the average R value for the 41 examined cases (Fig. 5, R=0.77±0.109). The stratification of the samples on the basis of the presence (25/41) or absence (16/41) of cirrhosis as the background liver disease changed the R values. In particular, the R value in the cirrhosis subgroup was 0.535±0.0947 (p<0.0001), suggesting that miR-24 was downregulated in HCC with respect to cirrhotic PT tissue. The R value in the non-cirrhotic liver subgroup was 1.137±0.211, suggesting no variation in the expression level (Fig. 5).

**Figure 3.**
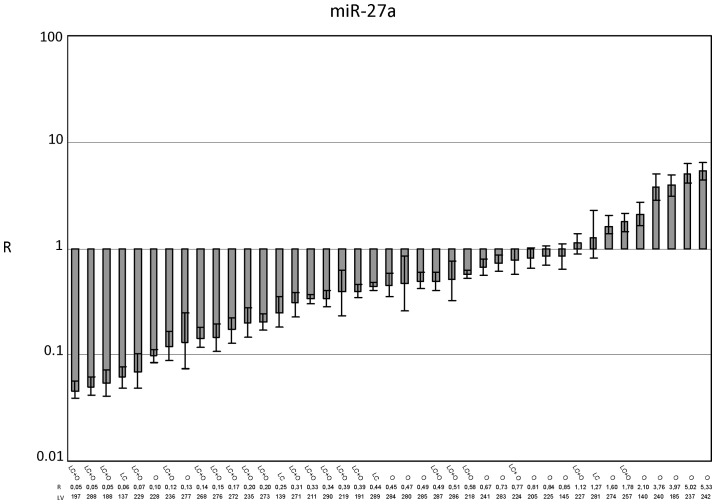
miR-27a expression levels detected by real-time PCR in tissues from biopsy specimens from patients affected by HCC. The graph indicates the R (RQ_HCC_/RQ_PT_) values of the human samples tested. The histograms are ordinated by increasing R. The background diseases are also indicated (LC, liver cirrhosis; O, other background disease i.e., B/C viral hepatitis, steatosis). The R values and the case number (LV) are listed under the graph.

Similar to miR-24, miR-27a (Figs. 3 and 5) did not show dysregulation among the 41 cases, as the average R value was 0.915±0.204. In the cirrhosis subgroup, the R value of 0.408±0.084 (p<0.0001) underlines the downregulation of miR-27a in HCC with respect to cirrhotic PT tissues. In the noncirrhotic subgroup, the R value was 1.707±0.455, indicating an upregulation of miR-27a expression. This result, however, was not statistically significant (Fig. 5).

**Figure 4.**
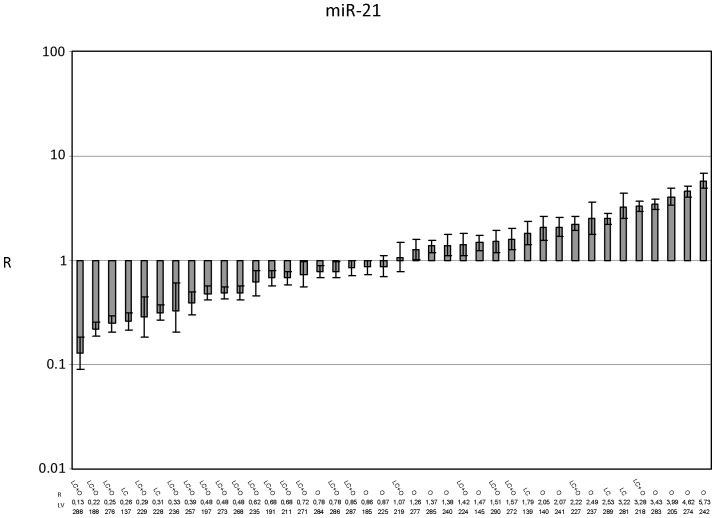
miR-21 expression levels detected by real-time PCR in tissues from biopsy specimens from patients affected by HCC. The graph indicates the R (RQ_HCC_/RQ_PT_) values of the human samples tested. The histograms are ordinated by increasing R. The background diseases are also indicated (LC, liver cirrhosis; O, other background disease i.e., B/C viral hepatitis, steatosis). The R values and the case number (LV) are listed under the graph.

**Figure 5.**
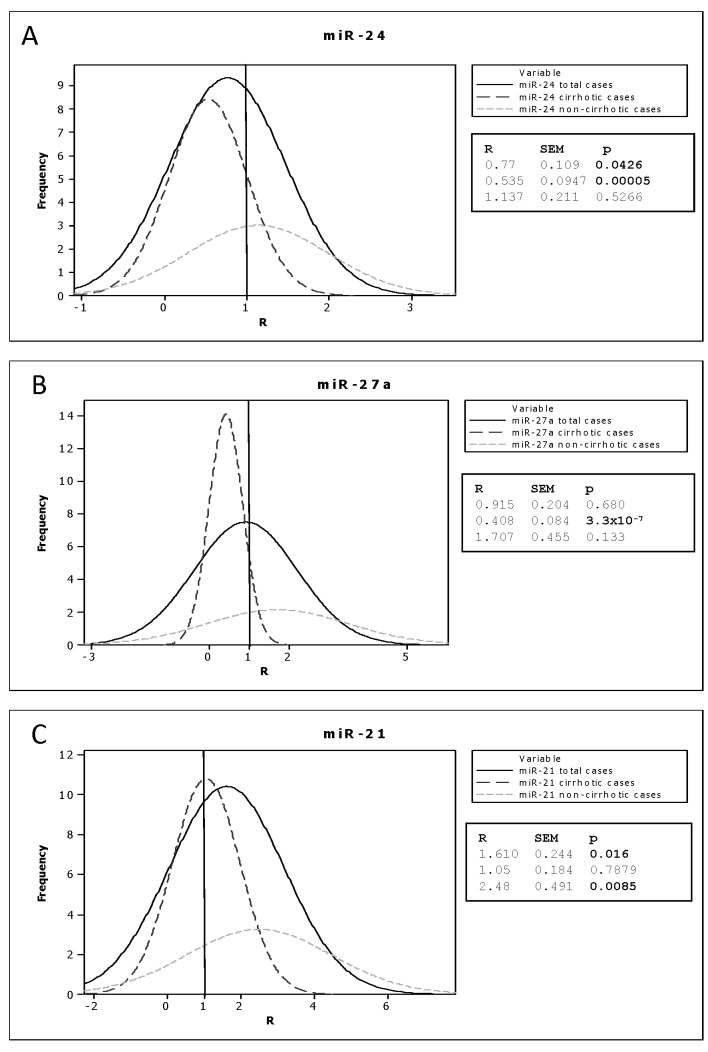
Normal distribution of the R values (RQ_HCC_/RQ_PT_) of miR-24 (A), miR-27a (B) and miR-21 (C) detected by real-time PCR in tissues from biopsy specimens from patients affected by HCC. The black curve indicates the normal distribution of R in all cases tested, the dashed black and gray curves refer to the HCC samples with, respectively, the presence or absence of liver cirrhosis as background disease.

For miR-21 (Fig. 4), the average R value was 1.610±0.244 (p=0.016), indicating the upregulation of miR-21 expression in HCC tissues with respect to PT tissues. The R value in the presence of cirrhosis was 1.05±0.184, and the R value in its absence was 2.48±0.491 (p=0.0085) (Fig. 5). This result suggested that miR-21 expression was unchanged in HCC that developed in cirrhotic livers but was increased in HCC that developed in noncirrhotic livers.

To determine whether the presence of the hepatitis virus influenced miR expression levels, we stratified the cirrhotic HCCs with respect to HBV/HCV infection status and we calculated the mean R values for each miR considered (Table IV). The patient clinical information concerning HBV/HCV infections and overall survival (OS) was available in 18 of 25 cases of cirrhosis.

For miR-24, a significant decrease in expression was observed in the HCV and HBV/HCV subclasses (R=0.523, p=0.0184; R=0.462, p=0.0311 respectively). No changes in expression levels were observed for the HBV and −/− subclasses. The mean R values directly correlated with the OS, expressed in months (Pearson correlation coefficient=0.988, p=0.00945). The mean OS rates were 59.5, 44.3, 39.7 and 57.5 months for the subclasses HBV, HCV, HBV/HCV and −/−, respectively.

For miR-27a, a significant decrease in expression was detected in the HBV, HCV and HBV/HCV subclasses (R=0.302, p=0.0084; R=0.412, p=0.0024; R=0.35, p=0.0234, respectively). No change in the expression level was observed for the −/− subclass (R=0.817).

No miR-21 expression level variation was detected among the 4 classes considered: HBV, HCV, HBV/HCV or no infection (−/−). In fact, the R values ranged from 0.7 to 1.3 with the exception of the −/− class, which displayed a slight increase in miR-21 expression in comparison to the other samples (R=1.41).

### Identification of a novel miRNA by bioinformatic tools and the verification of its expression by northern blotting

**Figure 6.**
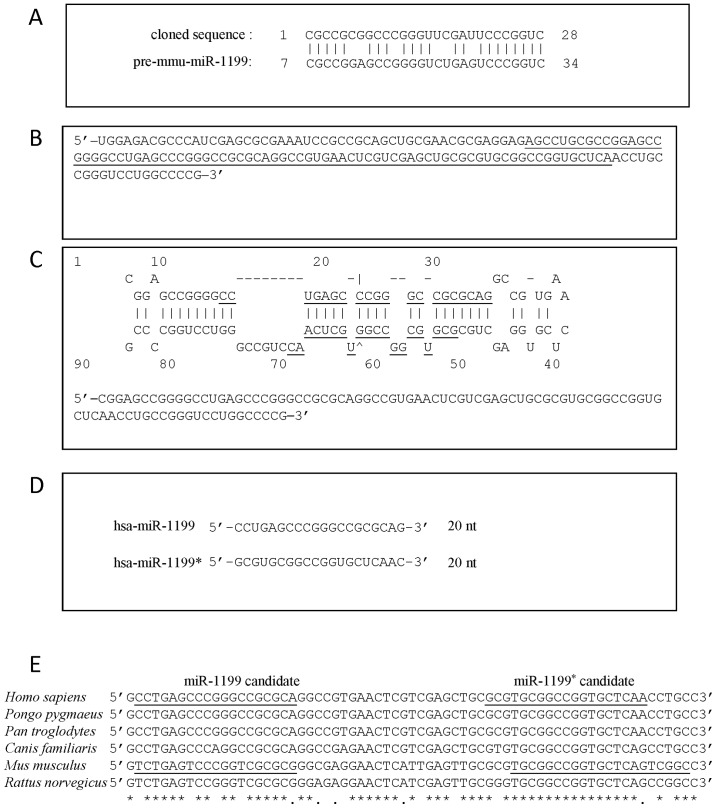
(A) Alignment of the human sequence cloned in the HA22T/VGH small expression library with the pre-mmu-miR-1199 (nt 7–34; miRBase accession no. MI0006307). (B) mFOLD input sequence corresponding to the human sequence obtained by BLAT analysis of the mouse sequence coding the mmumiR-1199 against the human genome. The part underlined is homologous to the mouse pre-miR-1199 sequence. (C) Predicted precursor hairpin structure of the input sequence indicated in B. The RNA secondary structure prediction was carried out using mFOLD version 3.2. The putative miRNA sequences are underlined. (D) The putative human miR-1199 and miR-1199^*^ sequences are indicated. (E) Alignment of the candidate microRNA-1199 sequences with mammalian genomes. The region is well conserved among the mammalian species indicated.

**Figure 7.**
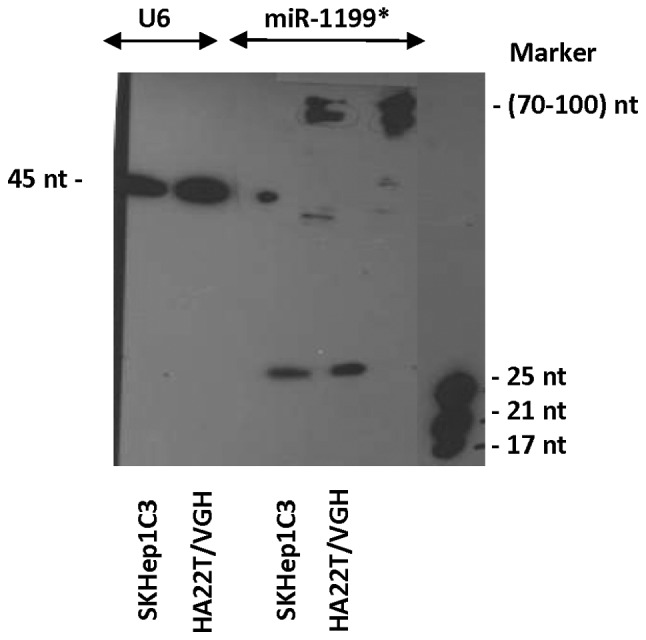
Northern blot analysis of the putative hsa-miR-1199^*^ expression. The mature miR-1199^*^ was detected in the HCC cell lines SKHep1C3 and HA22T/VGH. It was also detectable as the precursor form (70–100 nt) of the miR-1199^*^. The expression of the small U6 RNA (45 nt) used as loading control is also shown.

We cloned a sequence of 36 nucleotides that was homologous to a repeating element on chromosomes 1 and 6 as indicated by a matching of the sequence against the human genome (BLAT). However, if one performs an alignment of the sequence against the stem-loop sequences present in the miRBase database (http://www.mirbase.org) it is partially homologous (78%) to the precursor of the Mus musculus miR-1199 (Fig. 6A). To our knowledge, the human miR orthologue of mmu-miR-1199 has not been yet discovered, as it is not present within the miRBase official registry. The sequence coding for mmu-miR-1199 is located on chromosome 8 between the Prkaca and Rln3 genes. Based on an analysis of the synteny between mice and humans (www.ensembl.org), the corresponding homologous human sequence (79 nucleotides) is located on chromosome 19p13.2 in the 2nd exon of a 1424 nucleotide-long transcript (ENST00000269720) coding an uncharacterized 361 aa protein (ENSP00000269720). The human sequence (79 nucleotides) plus an additional 72 nucleotides flanking the 5′ and 3′ ends (Fig. 6B) is predicted to form a hairpin secondary structure, as evidenced by mFOLD 3.2 (Fig. 6C) and the sequence is well conserved among different mammalian species (Fig. 6E). Fig. 7 shows the expression of the novel candidate miR, as detected by northern blot analysis using biotinylated probes. The expression of the miR, here denominated hsa-miR-1199^*^ (* indicates the strand that matures from the 3′ arm of the candidate pre-miRNA), was evaluated in 2 HCC cell lines, HA22T/VGH and SKHep1C3. The mature form (∼22–25 nucleotides) and the precursor form (∼70–100 nucleotides) were clearly detectable. The strand that matures from the 5′ arm of the candidate pre-miRNA was not detected in the cell lines studied (data not shown).

## Discussion

In the present study we developed a small RNA expression library in the HCC cell line HA22T/VGH to identify new miRNAs and to study the profile of the expressed miRNAs. Among the 200 bacterial clones sequenced, 118 clones corresponded to 31 known miRs cloned with different frequencies and the miR-24, miR-27a, miR-21 were cloned with the highest frequency. Some of the 31 miRNAs cloned have been described previously to play a key role in cancer pathogenesis. miR-17, miR-19b, miR-20a and miR 92a belong to the cluster miR-17-92 known to be upregulated in several solid tumors (16,17). The miR-25 and miR-93 belong to the miR-106b-93-25 cluster that acts as oncomiRs (18). The miR-21 is well studied. It is commonly upregulated in several types of malignancies, included HCC (19–22). The miRs let7a and miR-7i belong to the let-7 family that is involved in apoptosis induction and inhibition of tumorigenicity (23–25). The miR-29b regulates epigenetic changes and triggers apoptosis and loss of tumorigenicity. It is downmodulated in colon and lung cancer, cholangiocarcinoma and in chronic lymphocytic leukemia (26,27). The miR-34b is a member of the miR-34 family known to be implicated in inhibition of the aggressive properties in several tumor cell lines by targeting MET, bcl2 and CDK4/6 (4). Although miR-122 has been identified as the most abundant liver-specific microRNA, clones carrying the miR-122 were not observed in the HA22T/VGH library. This may be due firstly to evidence that miR-122 is either silent or expressed at a very low level in most HCCs and transformed cell lines (28). Secondly, the sensitivity of the technique may have resulted in its lack of identification.

To date, few studies have been published concerning small RNA expression libraries in liver or in HCC cells/tissues. Fu *et al*(29) cloned 27 different miRs in a human fetal liver identifying 5 novel miRs. Among the most abundant, miR-24 was noted. Mizuguchi *et al*(30) cloned miRs in HCC and adjacent normal liver tissues by conventional cloning and 454 sequencing and they identified 7 novel miRs and several miR sequence modifications. As corroborated by our results, they found that miR-21 was expressed frequently as the edited form.

Considering the results obtained from a small RNA expression library in the HPV16^+^ cervical cancer cell line CaSki (31), with the identification of 46 different miRs among a total of 174 clones sequenced, notably several miRs cloned were the same found in our study. In particular, the miRs cloned with the highest frequency were miR-21, miR-27a and miR-24 as noted in our study. These and our findings may indicate a relevant role of miR-21, miR-24 and miR-27a in the malignant behavior of cervical cancer and HCC cell lines; for this reason we monitored their expression levels in human HCC tissues and their PT counterparts and then matched the miR expression levels with clinical patient features.

miR-24 and miR-27a displayed the same expression trend in 66.7% of the cases examined; this may have occurred due to the fact that they are clustered in 1 transcript on chromosome 19. To our knowledge, few data exist concerning miR expression during the progression from normal liver to HCC through cirrhosis. In this context, considering the subclass of HCC tumors developed in cirrhotic liver, miR-24 and miR-27a were downregulated in HCC in respect to PT tissues. This suggests that downregulation of miR-24 and miR-27a influences the hepatocyte transformation of cirrhotic tissues. Our data revealed miR-24 and miR-27a dysregulation in HCC in respect to their corresponding PT tissues and distinguished a profile in cirrhotic but not in non-cirrhotic tissues. For miR-24 in cirrhotic HCCs, the data obtained indicate a linear correlation between mean overall survival and miR-24 expression. The subclass of cirrhotic HCC with HBV/HCV infection displayed the worst outcome. It has been confirmed that miR expression can vary in response to HBV/HCV virus infections and their combination but the mechanisms are still poorly investigated.

miR-27a modification was different in cases without cirrhosis (but with other background diseases). In this tumor subclass miR-27a was upregulated in HCC tissues with respect to PT. It has been reported that a given miR may act as an oncomiR or tumor suppressor or that it may display pleiotropic properties in different cells, tissues or stages of cancer progression due also to the fact that a miR can control several targets (32). miR-27a was found to function as a tumor suppressor by targeting the anti-apoptotic protein FADD in human embryonic kidney cells (33). On the contrary, it acted as an oncomiR favoring the MDR1 (multi-drug resistance 1) protein upregulation in human ovarian cancer cells (34). miR-24 was described as an anti-oncomiR by regulating c-myc and E2F2 in the HCC-derived cell line HepG2 and causing inhibition of cell proliferation (35). In another study miR-24 acted as an oncomiR negatively regulating p16 in cervical carcinoma cells and the pro-apoptotic FAF1 protein in prostate, gastric and HeLa cancer cells (36,37). More studies are necessary to better explore the biological role of miR-24 and miR-27a in HCC and in other cancers. The observation that miR-24 expression is correlated with OS of cirrhotic HCC patients will be further validated in a larger group of patients.

miR-21, a well known wide-broad oncomiR, known to be overexpressed in HCC vs. normal liver was upregulated in HCC respect to PT tissues, and this became more evident in the subclass of HCC developed in non-cirrhotic liver. In the subclass of cirrhotic HCC the average R value was very near to 1 indicating no differences in the miR-21 expression level between cirrhotic PT and HCC tissues, thus supporting the hypothesis that miR-21 upregulation may be an early event during hepatocarcinogenesis. This is in line with the consideration that the tumor suppressor PTEN, one of the miR-21 targets, is already downmodulated in the pathological stages that often preceed the onset of HCC such as steatosis and cirrhosis (21). As a novel findings, our present data revealed miR-21 overexpression in HCC without cirrhosis as a background disease and found no expression variation between HCC and PT cirrhotic tissues (30). Jiang *et al*(38) reported miR-21 upregulation when the tumor tissue expression data were compared with the adjacent PT tissues, that were not stratified according to the background disease.

Another novel finding of the present report is the identification of a new human miR. During the sequence analyses we identified a 36-nt sequence that was partially homologous to the Mus musculus pre-miR-1199 and we realized that the corresponding human miR ortholog was not yet annotated in the miR registry. Three annotation criteria (expression and biogenesis criteria) (10) based on bioinformatic tools and experimental validation confirmed that miR-1199 was a new hsa-miR.

The expression analysis verified by northern blot analysis conducted in two human HCC cell lines showed that the expressed miR was miR-1199^*^ contained in the 3′ arm of the candidate hairpin-precursor miR. In contrast, in the mouse the miR strand cloned with the highest frequency was that matured from the the 5′ arm of the pre-miR-1199. However, to date no information is available concerning the mmu-miR-1199 role and target validations and further studies are necessary to identify them both in the human and mouse.

The annotation criteria also suggest that close homologs in other species can be annotated as miR orthologues without experimental validation, based on the criterion concerning the phylogenetic conservation of the ∼22 nt miR sequence and its predicted fold-back precursor secondary structure. In our case, miR-1199 may be annotated as a new miR also in *Pongo pygmaeus*, *Pan troglodytes*, *Canis familiaris* and *Rattus norvegicus*.

In conclusion, we identified the new human miR-1199, also phylogenetically conserved in other mammalian species. We also found that miR-24 and miR-27a were downregulated in HCC cancer developed in cirrhotic liver. Thus, these findings may contribute to advances in the discovery and validation of novel early molecular biomarkers of HCC progression from cirrhosis to cancer.

## Figures and Tables

**Table I.** Clinical and pathological characteristics of the studied population.

Case	Gender	Age	Grade	TNM	Background disease	HBV	HCV
137	M	69	G3	T3bN0M0	Active cirrhosis	NA	NA
139	M	65	G2	T2N0M0	Active cirrhosis	+	+
140	M	69	G2	T1N0M0	Aspecific reactive hepatitis	−	+
145	M	65	G2	T1N0M0	Steatotic hepatitis with portal and periportal fibrosis	NA	NA
185	M	66	G1	T1N0M0	Mildly active chronic hepatitis with steatosis of moderate level	+	−
188	M	73	G1	T3bN0M0	Cirrhosis with active chronic hepatitis	+	+
191	F	63	G1	T1N0M0	Cirrhosis with active chronic hepatitis	−	+
197	M	70	G2	T1N0M0	Cirrhosis with microvesicular and macrovesicular steatosis	−	−
205	M	73	G2	T1N0M0	Active chronic hepatitis	−	+
211	M	51	G2	T1N0M0	Cirrhosis with active chronic hepatitis with foci of macrovesicular steatosis and presence of iperplastic and regenerative macronodules	+	+
218	M	64	G2	T2N0M0	Cirrhosis with active chronic hepatitis	+	−
219	M	57	G1	T1N0M0	Cirrhosis with active chronic hepatitis	+	−
224	M	55	G3	T3bN0M0	Cirrhosis with active chronic hepatitis	+	+
225	M	49	G3	T3bN0M0	Microvesicular steatosis; focal lipofuscinosis; cholestasis	−	−
227	F	72	G2/G3	T1N0M0	Cirrhosis with active chronic hepatitis	−	+
228	M	59	G2	T1N0M0	Active chronic hepatitis of severe level with necrosis and bridging porto-portal fibrosis (HBsAG)	+	−
229	F	79	G2/G3	T3bN0M0	Cirrhosis with active chronic hepatitis	NA	NA
235	F	82	G3	T2N0M0	Cirrhosis with active chronic hepatitis	−	+
236	F	76	G1	T1N0M0	Cirrhosis with active chronic hepatitis	−	+
237	M	68	G2/G3	T1N0M0	Mildly active chronic hepatitis	−	+
240	M	71	G3	T3bN0M0	Active chronic hepatitis with necrosis and fibrosis ponte-portale	+	−
242	F	63	G2	T2N0M0	Active chronic hepatitis with focal and bridging porto-portal fibrosis	−	+
241	F	38	G2	T3N0M0	Reactive hepatitis	+	−
257	M	69	G1/G2	T1N0M0	Cirrhosis and hemochromatosis	−	−
268	F	68	G1	T1N0M0	Cirrhosis with active chronic hepatitis	+	−
271	F	71	G2/G3	T1N0M0	Cirrhosis with active chronic hepatitis	−	+
272	M	65	G1	T2N0M0	Cirrhosis with active chronic hepatitis and macrovesicular and microvesicular steatosis (30% of parenchyma)	−	−
273	M	73	G2	T1N0M0	Cirrhosis with active chronic hepatitis and mild macrovesicular and microvesicular steatosis	−	+
274	F	81	G2	T1N0M0	Mildly active chronic hepatitis with microvesicular and macrovesicular steatosis (30% of parenchyma)	NA	NA
276	M	72	G2	T1N0M0	Cirrhosis with active chronic hepatitis	−	+
277	F	75	G2	T2N0M0	Mildly active chronic hepatitis	−	−
280	F	74	G2/G3	T1N0M0	Mildly active chronic hepatitis	−	+
281	M	74	G2/G3	T3N0M0	Cirrhosis	−	−
283	M	78	G2	T1N0M0	Mildly active chronic hepatitis	+	−
284	M	76	G2	T1N0M0	Active chronic hepatitis	+	−
285	M	77	G2	T2N0M0	Active chronic hepatitis with necrosis ponte-portal of moderate/severe level	−	+
286	M	69	G3	T4N0M0	Active cirrhosis	−	+
287	M	63	G2	T2N0M0	Active cirrhosis	NA	NA
288	F	64	G2	T1N0M0	Active cirrhosis	−	−
289	M	75	G2	T1N0M0	Cirrhosis with iperplastic-displastic macronodules	−	+
290	M	65	G2/G3	T1N0M0	Active cirrhosis	+	−

NA, data not available. Age, in years.

**Table II.** Composition of the small RNA population cloned in the HA22T/VGH library.

RNA class	No.
miRNA	118
miRNA stem-loop^a^	4
Partially homologous miRNAs^b^	3
mRNA	10
rRNA	8
snRNA	2
Y RNA	1
Match with genome^c^	3
Mitochondrial	1
Empty vector	31
Uncorrected sequencing	19
Total	200

a Sequences that are not homologous to mature miRNAs but match with a miRNA hairpin precursors.

b Sequences that are partially homologous to mature miRNAs.

c Sequences that do not form miRNA-specific hairpin precursors but match with genomic sequences.

**Table III.** List of the known miRNAs identified in the library.

miRNA	Frequency	Genomic locus (*Homo sapiens*)
hsa-miR-let7a	1	9q22.31
		11q24.1
		22q13.31
hsa-miR-let7i	2	12q14.1
hsa-miR-10a	1	17q21.31
hsa-miR-17	1	13q31.3
hsa-miR-19b	1	13q31.3
		Xq26.2
hsa-miR-20a	2	13q31.3
hsa-miR-21	51	17q22
hsa-miR-22	2	17p13.3
hsa-miR-23a	2	19p13.2
hsa-miR-24	7	9q22.32
		19p13.2
hsa-miR-25	1	7q22.1
hsa-miR-26a	3	3p22.2
		12q13.2
hsa-miR-26b	2	2q35
hsa-miR-27a	17	19p13.2
hsa-miR-27b	1	9q22.32
hsa-miR-29b	1	7q32.2
		1q32.1
hsa-miR-30b	3	8q24.22
hsa-miR-30c	1	1p34.2
		6q13
hsa-miR-30e	1	1p34.2
hsa-miR-34b	1	11q23.1
hsa-miR-92a/b^a^	1	92a: 13q31.3; Xq26.2
		92b: 1q22
hsa-miR-92a	3	13q31.3
		Xq26.2
hsa-miR-93	1	7q22.1
has-miR-99a	1	21q21.1
hsa-miR-181a	2	1q31.3
		9q33.3
hsa-miR-224	1	Xq28
hsa-miR-324-5p	1	17p13.1
hsa-miR-365	3	16p13.12
		17q11.2
hsa-miR-424	2	Xq26.2
hsa-miR-1308	1	Xp22.11
hcmv-mir-US25-2	1	Genoma CMV

a The sequence cloned did not allow the discrimination between the form a and the form b of miR-92.

**Table IV.** R values in cirrhotic HCCs subclassified in respect to hepatitis viral infections and the correlations between R values and overall survival.

	HBV (n=3)		HCV (n=7)		HBV/HCV (n=4)		−/− (n=4)		Pearson correlation (R vs OS)	
				
	R value (mean)	p-value	R value (mean)	p-value	R value (mean)	p-value	R value (mean)	p-value	Correlation coefficient	p-value
miR-24	0.707	0.554	0.523	**0.0184**	0.462	**0.0311**	0.645	0.276	0.988	**0.00945**
miR-27a	0.302	**0.0084**	0.412	**0.0024**	0.35	**0.0234**	0.817	0.694	0.373	0.694
miR-21	1.285	0.705	1.155	0.74	1.027	0.72	1.41	0.57	0.406	0.724

OS, overall survival. The bold numbers refer to significant p-values.
